# Case Report: Surgical Treatment for Intranasal Meningoencephalocele in a Cat

**DOI:** 10.3389/fvets.2020.00532

**Published:** 2020-08-21

**Authors:** Yuta Nozue, Midori Yamazaki, Kohei Nakata, Yukiko Nakano, Go Yuki, Arata Kimata, Hiroaki Kamishina

**Affiliations:** ^1^The Animal Medical Center of Gifu University, Gifu University, Gifu, Japan; ^2^The United Graduate School of Veterinary Sciences, Gifu University, Gifu, Japan; ^3^Kimata Animal Hospital, Hamamatsu, Japan; ^4^Center for Highly Advanced Integration of Nano and Life Sciences, Gifu University, Gifu, Japan

**Keywords:** meningoencephalocele, surgical treatment, graft, tunica vaginalis, cribriform plate reconstruction, case report

## Abstract

A 4-month-old cat with epileptic seizures and nasal discharge was presented, and diagnosed with intranasal meningoencephalocele based on the clinical symptoms and findings of CT and MRI. As liquorrhea was suspected, the meningoencephalocele was surgically excised and the postoperative course was favorable. For cribriform plate reconstruction, the autologous tunica vaginalis was used. Rhinorrhea of the cerebrospinal fluid and seizures disappeared after surgery. On CT and MRI at 7 months after surgery, septum formation between the cranial and nasal cavities was confirmed. Currently, no seizures have occurred even though no oral antiepileptic agent was administered. There are few reports of surgical treatment for meningoencephalitis, and there are no reports of reconstruction of the cribriform plate using tunica vaginalis, so we reported the details.

## Introduction

Meningoencephalocele is a congenital disease in which a part of the cranial content extracranially prolapses through a skull defect. Prolapse of the meninges alone is termed meningocele, and prolapse of the meninges and cerebral parenchyma is termed meningoencephalocele (1). In humans, meningoencephalocele is classified into that in the occipital, frontoethmoidal, cranial base, or cranial convexity region based on the location of the skull defect, and these are further subdivided (2). Development of meningoencephalocele in the occipital region accounts for ~80% of cases in humans (3), and development in the other regions is rare. When meningoencephalocele is present in the nasal cavity, it is difficult to find visually, and is discovered when respiratory disturbance, snoring, liquorrhea, and repeated meningitis are observed (1, 4).

In the small animal field, only a few reports on meningoencephalocele are available, and limiting to surgically treated cases, only a dog with intranasal meningoencephalocele (5) and a cat with parietal meningoencephalocele (6) have been reported.

## Case Presentation

A 4-month-old, non-castrated male Japanese cat weighing 3.0 kg was presented. Focal seizures causing facial muscle spasms and salivation as the main symptoms appeared from 4 months of age, and the cat was brought to our hospital. This symptom was seen about once every 2 weeks, and there were no signs of pre- or post-ictal sign. No loss of consciousness was noted during the seizure, and the seizure subsided in <1 min. The first examination day was designated as the first hospital day. Serous nasal discharge was noted on physical examination, but no change was observed in outward appearance. No abnormality was detected on the blood test, blood chemistry, thoracoabdominal radiography, or abdominal ultrasonography. Blood chemistry test was performed including those that could cause seizures (Ca, Na, Glu, NH3, TBA, BUN, CREA). Excluding seizures as a neurological manifestation, no abnormality was noted on the neurological examination. Gabapentin at 8.3 mg/kg, BID, was administered (BID was adopted because TID administration caused mild ataxia) and the symptoms improved. Tonic-clonic seizures that last 2–3 min developed daily from the 27th hospital day and seizure symptoms progressed, for which oral administration of phenobarbital at 2 mg/kg, BID, was initiated. Based on the case signalment and progressive clinical course, differential diagnosis included a congenital disease, inflammatory disease, and degenerative disease. On the 36th hospital day, MRI and CT were performed to closely examine the intracranial disease.

**Figure 1:**
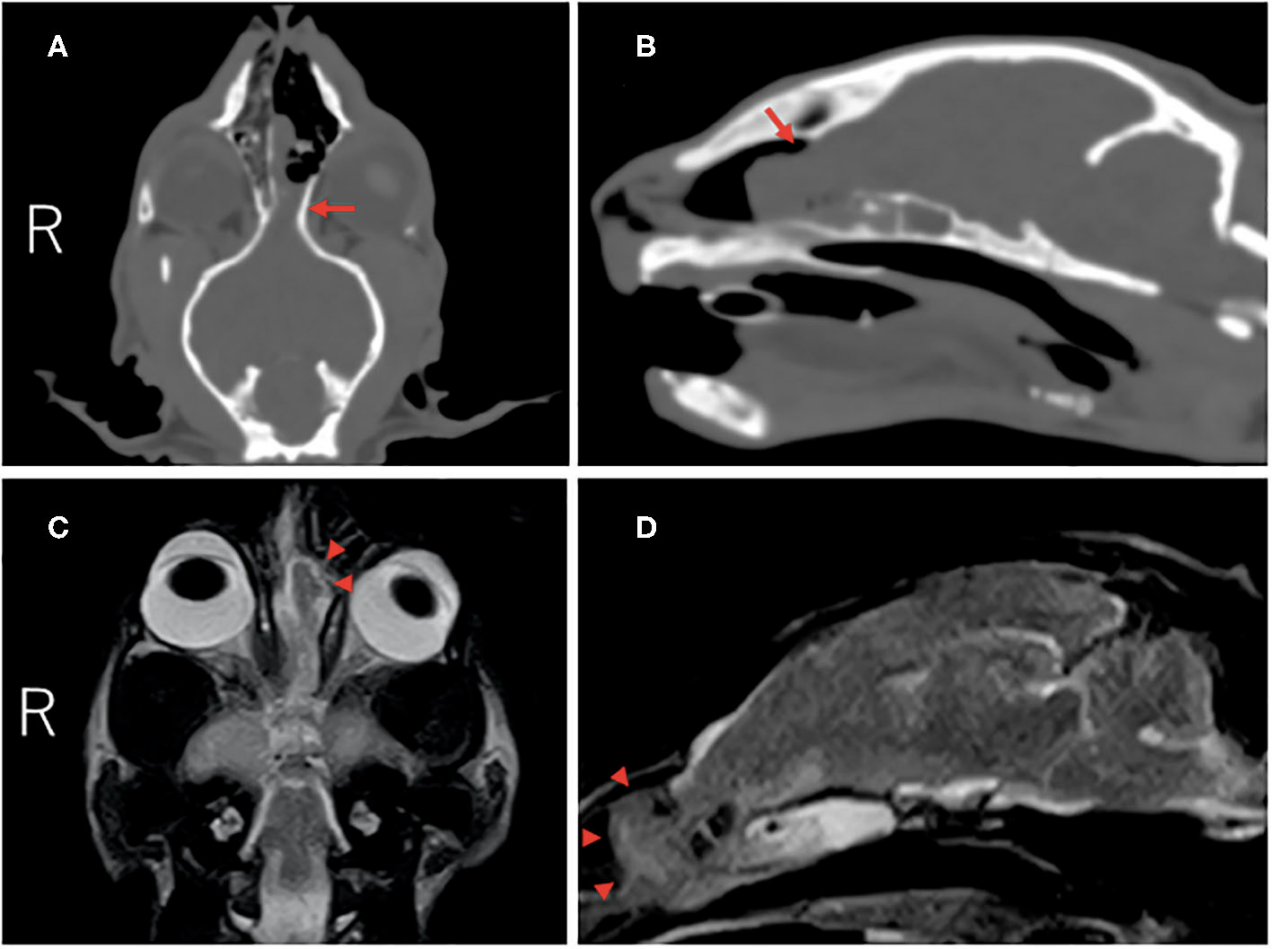
CT and MR images of the cranium and brain at diagnosis. Dorsal **(A)** and parasagittal **(B)** CT images showed the defect of the left cribriform plate (red arrow). On dorsal **(C)** and parasagittal **(D)** T2-weighted MR images, the left olfactory bulb protruded into the nasal cavity through the defect (red arrowheads).

On CT (Alexion, Toshiba, Tochigi, Japan), a defect of the left cribriform plate and rightward deviation of the nasal septum were observed, and an iso-dense mass lesion with cerebral parenchyma occupied the nasal cavity. This mass lesion was continuous with the cerebral parenchyma (Figures 1A,B). On MRI (0.4-Tesla APERTO Eterna, Hitachi, Tokyo, Japan), a lesion continuous with the cerebral parenchyma occupying the nasal cavity exhibiting iso-high intensity on T2-weighted imaging (TE: 100 ms, TR: 4,500 ms), iso-high intensity on FLAIR (TE: 100 ms, TR: 9,000 ms), and iso-intensity on T1-weighted imaging (TE: 13 ms, TR: 350 ms) was noted (Figures 1C,D). This region was mildly enhanced with gadolinium (OMNISCAN INTRAVENOUS INJECTION, Daiichi Sankyo, Tokyo, Japan) intravenous administration. In the sphenoid sinus, an occupied structure exhibiting high intensity on T2-weighted imaging was observed. No other cerebral parenchymal lesion or ventricular enlargement was observed. The cerebellum was slightly displaced toward the caudal side. No CSF test was performed in consideration of the risk of cerebellar herniation.

The mass lesion was diagnosed as intranasal meningoencephalocele because the animal was young and developed progressive seizure symptoms, and prolapse of the cerebral parenchyma into the nasal cavity was suggested by CT and MR imaging.

**Figure 2:**
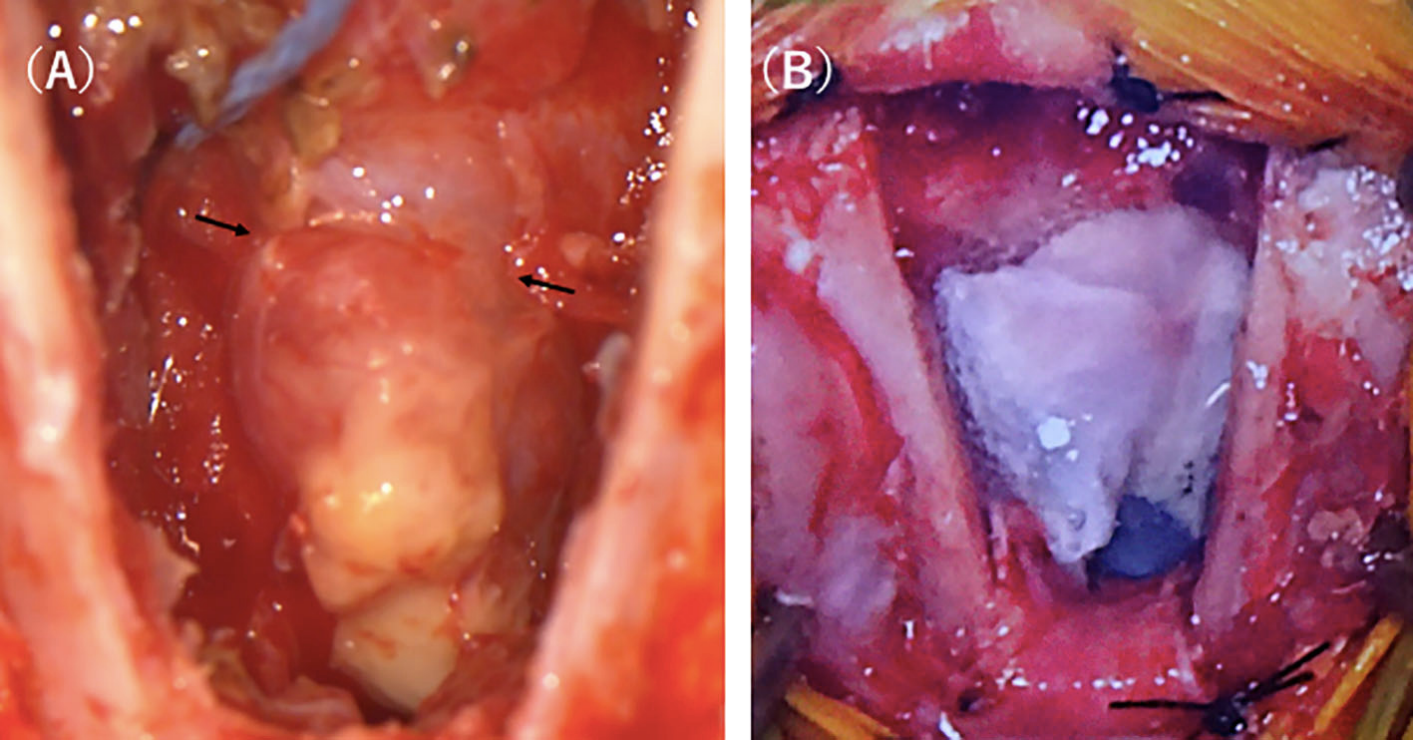
Intraoperative photographs of the intranasal meningoencephalocele. After approaching the lesion by transfrontal craniotomy, a discolored mass connected to the brain was easily identified **(A)**. After resecting the mass the defect was covered with the sections of the tunica vaginalis which were further covered by fibrin glue **(B)**.

In the present case, concomitant development of bacterial meningoencephalitis was expected because the left cribriform plate was defective, suggesting that the cerebral parenchyma prolapsed into the nasal cavity, thereby causing liquorrhea as it was constantly exposed to the outside environment. In order to control the seizures, surgical resection of the encephalocele and reconstruction of the defective cribriform plate were recommended. Cephalexin at 20 mg/kg, BID, and enrofloxacin at 5 mg/kg, SID, were administered perioperatively and 1 week after surgery. Anesthesia was induced with propofol (PropoFlo28, Zoetis Japan, Tokyo, Japan) and maintained by isoflurane (Isoflurane for animals, Intervet, Tokyo, Japan) inhalation after tracheal intubation. During surgery, pain was managed with remifentanil (Ultiva Intravenous, Janssen Pharmaceutica, Tokyo, Japan), and blood pressure was managed with ephedrine (Ephedrin “NAGAI,” Nichi-iko Pharmaceutical, Toyama, Japan) and dopamine (Dominin for I.V.Infusion, Nippon Shinyaku Co., Kyoto, Japan). After the anesthesia was stabilized, the cat was placed in the prone position and disinfected. The cat was castrated before brain surgery, and the autologous tunica vaginalis was collected. After the scalp incision, craniotomy was carried out via the transfrontal sinus approach using a sagittal saw (CORE 2, Stryker Corporation, Michigan, United States). Bone tissue below the frontal bone was removed as much as possible using an ultrasonic aspirator (Sono Cure, TOKYO IKEN CO., Tokyo, Japan), and the lesion was approached. The right cribriform plate was confirmed, but the left cribriform plate was absent and a mass considered to be the cerebral parenchyma prolapsed from this region. The prolapsed mass was entirely yellow in color compared with normal brain tissue (Figure 2A). In addition, the perpendicular plate of the ethmoid was displaced to the right by the mass in the cribriform plate-defective region, and the mass subsided in this defective region compared with in other regions. Setting the border at this region, the mass was carefully cut using a bipolar knife (Force Triad, Covidien Japan, Tokyo, Japan). The excised lesion was subjected to histopathological examination. In addition, a swab of the encephalocele was subjected to a bacteriological test.

For cribriform plate reconstruction, the autologous tunica vaginalis and a liquid tissue adhesive, fibrin glue (BeriplastP Comibi-Set Tissue adhesion, CSL Behring K.K., Tokyo, Japan), were used. The tunica vaginalis excised before brain surgery was arranged to cover the defective region of the cribriform plate over the stump of the mass, and the liquid tissue adhesive was applied to the surrounding tissue (Figure 2B). Using a self-drilling titanium screw (Bone screw Cross-Pin Self-Drilling ϕ: 1.2 mm, L: 3 mm, Stryker Corporation, Michigan, United States) and titanium mesh (Dynamic Mesh Standard, Stryker Corporation, Michigan, United States), the cut frontal bone was fixed and closed.

**Figure 3:**
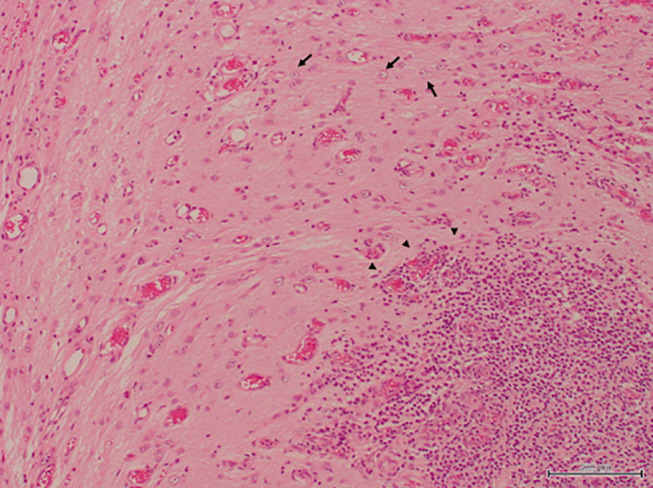
The resected specimen contained nerve tissues, leading to a diagnosis of meningoencephalocele. An increase in astrocytes and infiltration of inflammatory cells which consisted of many lymphocytes, plasmacytes, macrophages and a few neutrophils were recognized in the specimen (H&E stain, bar = 200 μm).

The excised specimen was immediately fixed with formalin and histopathologically examined (Figure 3). In HE-stained specimen, the submitted tissue contained a part of the cerebrum, mostly edematous cerebral parenchyma, and a region with a scarce neural network was present. Plasma cells, lymphocytes, macrophages, and a small number of neutrophils infiltrated with cicatrization by fibrosis or astrocyte proliferation. These inflammatory cells often infiltrated around the adjacent blood vessels. Degeneration and necrosis were intermittent, and calcification was noted in this region. Degenerated neurons were scattered, and the nucleus and cytoplasm were condensed. No bacteria or tumor cells were found. In the present case, liquorrhea was suspected and bacterial meningoencephalitis was considered possible, but no bacteria was also detected on the bacterial test.

After surgery, no seizures occurred and serous nasal discharge was resolved. Abnormality in the sense of smell was of concern because the olfactory bulb was resected, but no abnormality was observed and the cat had a good appetite. No paralysis or gait disturbance was exhibited. Neurological tests performed 1 week, 1, 3, and 7 months after surgery showed no abnormalities in consciousness level, gait, behavior, cranial nerve test, and postural response.

**Figure 4:**
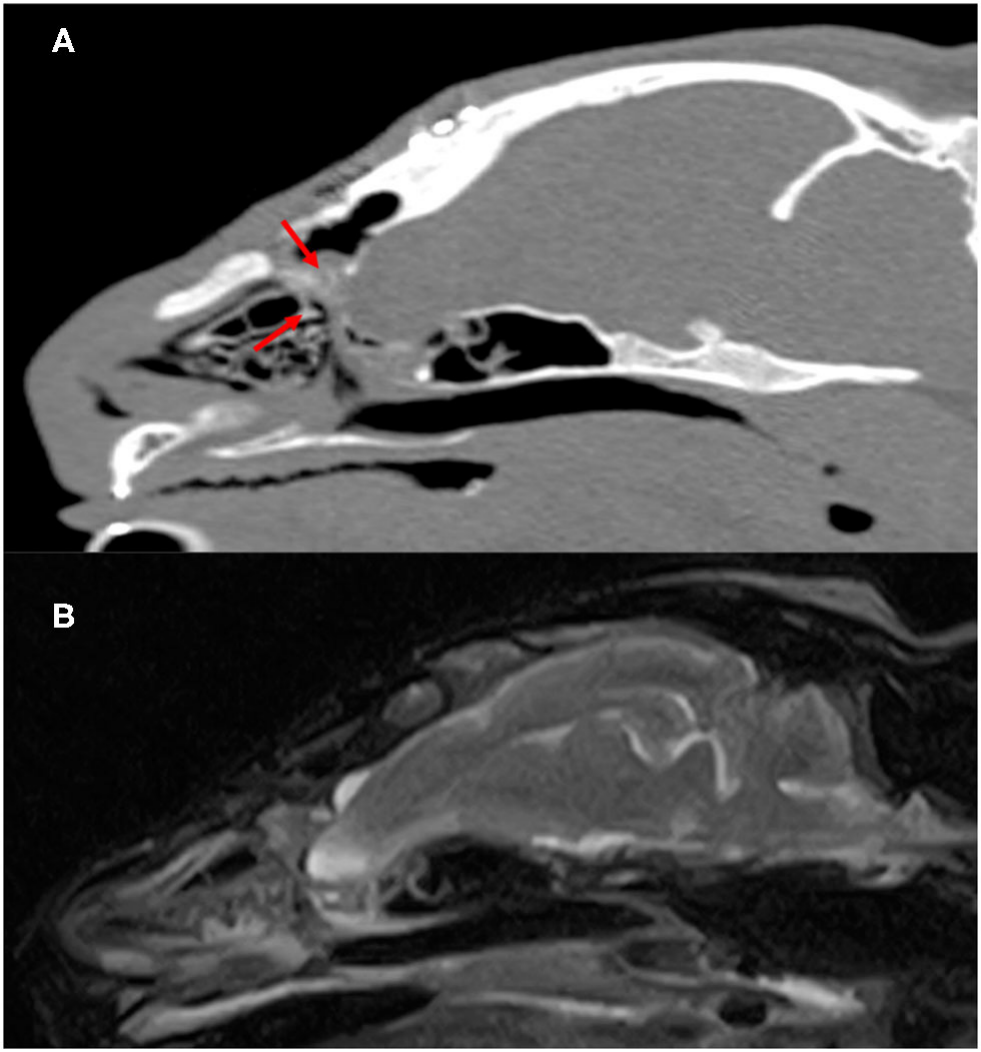
Postoperatve CT and MR images of the cranium and brain 7 months after surgery. Parasagittal CT images **(A)** showed slightly superabsorbent structures separate cranial cavity and nasal cavity (red arrow). On parasagittal T2-weighted MR images **(B)**, there is no apparent departure of the brain into the nasal cavity.

Seven months after surgery, the formation of the septum dividing the nasal cavity and brain were confirmed on MRI and CT (Figure 4). In addition, the structure occupying the sphenoid sinus that had been recognized preoperatively had disappeared. No seizures were noted after surgery. Therefore, we planned to gradually reduce antiepileptic drugs and stop the drug, but at the owner's discretion, the drug was continued for 6 months after the operation. However, no seizures were observed for 24 months since the drug was stopped.

## Discussion

Meningoencephalocele is a congenital disease in which the cranial content extracranially prolapses through a skull defect. No factor associated with its development has been clarified, but developmental abnormality of the neuroectoderm during pregnancy has been suggested (7).

It is diagnosed based on the combination of clinical signs, such as liquorrhea and neurological manifestations accompanying meningitis, and imaging findings (2). CT is useful to evaluate skull defects and MRI is useful to evaluate the content of the encephalocele (7). The definite diagnosis is made by histopathological examination of the excised specimen. The differential diagnosis of intranasal meningoencephalocele in humans includes glioma, intranasal polyp, and dermoid cyst (4). Hydrocephalus and other concomitant congenital abnormalities of the brain are considered to be prognostic factors (8, 9).

Jeffery reported that hemorrhage, malacia, degeneration of the white matter, and inflammatory cell infiltration were noted on pathological examination (10), similar with the pathological findings in our case. Adherent fibrous tissue was present on the surface of the excised encephalocele, and nerve degeneration and necrosis were scattered throughout the cerebral parenchyma. Inflammation and fibrosis were severe in this region, suggesting that the tissue was altered by physical compression in the defective region, causing seizures. In the present case, liquorrhea was suspected and bacterial meningoencephalitis was considered possible, but no bacteria was pathologically observed or detected on the bacterial test.

In general, surgery is the main treatment method for meningoencephalocele in humans (11). There are several surgical treatment methods that are used corresponding to the location of the skull defect (1). In the small animal field, only 2 cases of surgical treatment of meningoencephalocele have been reported (5, 6). In a report summarizing 22 cases of meningocele/meningoencephalocele in dogs, favorable outcomes were achieved by medical treatment for mild tissue protrusion (12). However, in the present case, liquorrhea was suspected, inflammation of encephalocele was suggested on MRI, and seizures repeatedly occurred, indicating the necessity of surgical treatment. In humans, surgical treatment is strongly recommended when respiratory disturbance, recurrent meningitis, progressive visual disorder, and liquorrhea are present (13). Thus, surgery was performed aiming at resection of the tissue that had prolapsed into the nasal cavity and septum formation to separate the brain from the outside. As the right cribriform plate was present, the prolapsed brain tissue was resected after setting as the boundary between the nasal cavity and the cranial cavity at this region. For septum formation, the autologous tunica vaginalis collected before brain surgery was used. In the veterinary surgery field, biological membranes, such as fascia, and artificial materials, such as artificial dura, may be used. The fascia is thin depending on the size of the animal, limiting the excisable size, and it lacks flexibility. On the other hand, the size of artificial dura can be changed as needed, but implant infection and foreign body reaction are possible. The tunica vaginalis is superior in flexibility and toughness, and can overcome problems described above. Its use for surgical reduction of perineal hernias and bladder wall replacement has been reported (14, 15), but its use for reconstruction of a skull defect in meningoencephalocele cases has not been reported. Although long-term follow-up is necessary, the liquorrhea was resolved after surgery and a structure that separates the nasal cavity from the cranial cavity was confirmed on CT images. Preoperative MR images showed that the structure occupying the sphenoid sinus had a high signal on T2-weighted imaging, suggesting that leaked cerebrospinal fluid had accumulated, but it was disappeared after surgery. These suggest that reconstruction of the defective region with the autologous tunica vaginalis was successful.

## Data Availability Statement

The original contributions presented in the study are included in the article/supplementary material, further inquiries can be directed to the corresponding author.

## Ethics Statement

Written informed consent was obtained from the owners for the participation of their animals in this study.

## Author Contributions

MY, KN, GY, AK, and HK helped with the diagnosis of this case and participated in clinical case management. HK participated in the review and editing the manuscript. All authors contributed to the article and approved the submitted version.

## Conflict of Interest

The authors declare that the research was conducted in the absence of any commercial or financial relationships that could be construed as a potential conflict of interest.

## References

[1] Abdel-Aziz M El-Bosraty H Qotb M El-Hamamsy M El-Sonbaty M Abdel-Badie H. Nasal encephalocele: endoscopic excision with anesthetic consideration. Int J Pediatr Otorhinolaryngol. (2010) 74:869–73. 10.1016/j.ijporl.2010.04.015 20554034

[2] Tirumandas M Sharma A Gbenimacho I Shoja MM Tubbs RS Oakes WJ. Nasal encephaloceles: a review of etiology, pathophysiology, clinical presentations, diagnosis, treatment, and complications. Childs Nerv Syst. (2013) 29:739–44. 10.1007/s00381-012-1998-z 23247827

[3] Mahajan C Rath GP Dash HH Bithal PK. Perioperative management of children with encephalocele: an institutional experience. J Neurosurg Anesthesiol. (2011) 23:352–6. 10.1097/ANA.0b013e31821f93dc 21633311

[4] Steven RA Rothera MP Tang V Bruce IA. An unusual cause of nasal airway obstruction in a neonate: trans-sellar, trans-sphenoidal cephalocoele. J Laryngol Otol. (2011) 125:1075–8. 10.1017/S0022215111001800 21791157

[5] Martle VA Caemaert J Tshamala M Van Soens I Bhatti SF Gielen I. Surgical Treatment of a Canine Intranasal Meningoencephalocele. Vet Surg. (2009) 38:515–9. 10.1111/j.1532-950X.2009.00534.x 19538674

[6] Dewey CW Brewer DM Cautela MA Talarico LR Silver GM. Surgical treatment of a meningoencephalocele in a cat. Vet Surg. (2011) 40:473–6. 10.1111/j.1532-950X.2011.00813.x 21418253

[7] Hoving E.W. Nasal encephaloceles. Childs Nerv Syst. (2000) 16:702–6. 10.1007/s003810000339 11151720

[8] Hoving EW Vermeij-Keers C. Frontoethmoidal encephaloceles, a study of their pathogenesis. Pediatr Neurosurg. (1997) 27:246–56. 10.1159/000121262 9620002

[9] Naidich TP Altman NR Braffman BH McLone DG Zimmerman RA. Cephaloceles and related malformations. Am J Neuroradiol. (1992) 13:655–90. 1566723 PMC8333224

[10] Jeffery N. Ethmoidal encephalocoele associated with seizures in a puppy. J Small Anim Pract. (2005) 46:89–92. 10.1111/j.1748-5827.2005.tb00299.x 15736816

[11] Baradaran N Nejat F Baradaran N El Khashab M. Cephalocele: report of 55 cases over 8 years. Pediatr Neurosurg. (2009) 45:461–6. 10.1159/000277622 20110760

[12] Lazzerini K Gutierrez-Quintana R Jose-Lopez R McConnell F Goncalves R McMurrough J Clinical features, imaging characteristics, and long-term outcome of dogs with cranial meningocele or meningoencephalocele. J Vet Intern Med. (2017) 31:505–12. 10.1111/jvim.14638 28247440 PMC5354015

[13] Franco D Alonso N Ruas R da Silva FR Franco T. Transsphenoidal meningoencephalocele associated with cleft lip and palate: challenges for diagnosis and surgical treatment. Childs Nerv Syst. (2009) 25:1455–8. 10.1007/s00381-009-0918-3 19506889

[14] Wongsetthachai P Pramatwinai C Banlunara W Kalpravidh M. Urinary bladder wall substitution using autologous tunica vaginalis in male dogs. Res Vet Sci. (2011) 90:156–9. 10.1016/j.rvsc.2010.05.015 20542305

[15] Pratummintra K Chuthatep S Banlunara W Kalpravidh M. Perineal hernia repair using an autologous tunica vaginalis communis in nine intact male dogs. J Vet Med Sci. (2013) 75:337–41. 10.1292/jvms.11-0478 23131842

